# Impact of Comorbid Prematurity and Congenital Anomalies: A Review

**DOI:** 10.3389/fphys.2022.880891

**Published:** 2022-07-01

**Authors:** Julia K. Gunn-Charlton

**Affiliations:** ^1^ Department of Paediatrics, Mercy Hospital for Women, Melbourne, VIC, Australia; ^2^ Heart Research Group, Murdoch Children’s Research Institute, Melbourne, VIC, Australia; ^3^ Department of Paediatrics, The University of Melbourne, Melbourne, VIC, Australia

**Keywords:** prematurity, congenital anomaly, neonate, brain injury, neurodevelopment

## Abstract

Preterm infants are more likely to be born with congenital anomalies than those who are born at full-term. Conversely, neonates born with congenital anomalies are also more likely to be born preterm than those without congenital anomalies. Moreover, the comorbid impact of prematurity and congenital anomalies is more than cumulative. Multiple common factors increase the risk of brain injury and neurodevelopmental impairment in both preterm babies and those born with congenital anomalies. These include prolonged hospital length of stay, feeding difficulties, nutritional deficits, pain exposure and administration of medications including sedatives and analgesics. Congenital heart disease provides a well-studied example of the impact of comorbid disease with prematurity. Impaired brain growth and maturity is well described in the third trimester in this population; the immature brain is subsequently more vulnerable to further injury. There is a colinear relationship between degree of prematurity and outcome both in terms of mortality and neurological morbidity. Both prematurity and relative brain immaturity independently increase the risk of subsequent neurodevelopmental impairment in infants with CHD. Non-cardiac surgery also poses a greater risk to preterm infants despite the expectation of normal *in utero* brain growth. Esophageal atresia, diaphragmatic hernia and abdominal wall defects provide examples of congenital anomalies which have been shown to have poorer neurodevelopmental outcomes in the face of prematurity, with associated increased surgical complexity, higher relative cumulative doses of medications, longer hospital and intensive care stay and increased rates of feeding difficulties, compared with infants who experience either prematurity or congenital anomalies alone.

## Introduction

**FIGURE 1:**
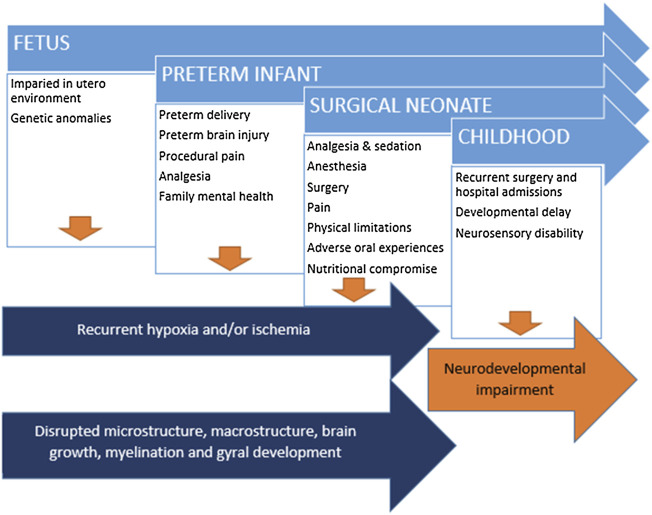
Disrupted brain development and brain injury in context of cumulative insults and risk factors subsequent developmental delay and neurodevelopmental impairment.

Congenital anomalies which require surgery during the neonatal period predispose infants to brain injury and subsequent neurodevelopmental impairment, including both cardiac and non-cardiac lesions (Snookes et al., 2010; Walker et al., 2012; Gaynor et al., 2015; Gunn et al., 2016a; Stolwijk et al., 2016; Moran et al., 2019). Contributing factors include the underlying anomaly and associated genetic contributors, the fetal environment before birth, intensive care exposure and the perioperative period including medications and the environment, surgery and anesthesia, prolonged hospitalization and the impact on the family and caregivers. Neonates born preterm are also prone to increased rates of brain injury and impaired neurodevelopment (van Bel et al., 2019; Cheong et al., 2021); this risk is markedly amplified with the frequent co-existence of prematurity and congenital anomalies, which is the focus of this review (Figure 1).

## Brain Injury in Preterm Infants

The fetal and preterm brain is vulnerable to injury throughout the third trimester, regardless of *in utero* or *ex utero* status. The impaired *in utero* environment may compromise brain growth and development due to placental dysfunction, uterine dysfunction, maternal ill health, infection, or injury (Ophelders et al., 2020). The preterm delivery of a fetus is usually due to one or more of these reasons. Conversely, an abnormality in the fetus can also lead to both preterm delivery and/or impaired brain growth and development. As such, preterm infants are vulnerable to brain injury and impaired development both due to the reasons for their prematurity, but also as a consequence of preterm delivery itself. Brain development is programmed throughout the third trimester, regardless of whether the fetus or neonate is *in utero* or *ex utero* (Buss et al., 2012). As such, the vulnerability of the brain to insult is likely to lead to a similar pattern of injury regardless of whether the baby is yet born. It is thus helpful to consider the fetal and neonatal brain on a continuum of growth and development with the same vulnerabilities and protective strategies in the face of hypoxia, ischemia, infection, toxins and over- or under-stimulation.

The more preterm an infant is at birth, the higher the risk of brain injury, such that every extra day *in utero* is of benefit in minimizing events leading to injury, as long as both the *in utero* environment and the fetus themself is healthy (Ophelders et al., 2020; Cheong et al., 2021). The typical pattern of brain injury experienced by preterm neonates is well described and includes intraventricular hemorrhage, venous infarction, periventricular echogenicity and leukomalacia, post-hemorrhagic ventricular dilatation, and hypoxic ischemic injury (Inder et al., 2021; Ortinau and Neil, 2015). However, only the more severe lesions evident on neuroimaging translate into longer term outcomes, with environmental factors exerting a greater influence on outcomes over time, especially in those with milder brain injury on imaging (Jansen et al., 2021). Furthermore, developmental delay in the preschool years does not necessarily portend later neurodevelopmental impairment in later years, particularly in the absence of neurosensory impairment or motor disability such as cerebral palsy (Hack et al., 2005).

## Prematurity and Congenital Anomalies

Prematurity and congenital anomalies frequently co-exist. In New York, congenital malformations were twice as likely in the preterm population and 3.5 times more likely in very preterm infants, for all systems other than pulmonary disease (Kase and Visintainer, 2007). Further, nationwide analysis of more than one million births confirmed these findings of a strong association between prematurity and congenital malformations (Egbe et al., 2015).

In 2015–2016, in Victoria, Australia, 1 in 22 pregnancies had a congenital anomaly, most commonly chromosomal anomalies, followed by urogenital, cardiac, musculoskeletal and nervous system problems (CCOPPM, 2018). Population level data confirmed that preterm infants were more likely than full-term infants to be born with a congenital malformation. Moreover, infants with congenital anomalies were 3 times more likely to be born preterm than those without. Furthermore, regardless of gestation, small for gestational age (SGA) was more common in infants with congenital anomalies and, regardless of gestation, congenital anomalies were more common in infants born SGA.

## Neurodevelopmental Delay Following Neonatal Surgery

Anomalies which require surgical management in the newborn period inevitably increase the risk of brain injury and subsequent neurodevelopmental impairment. Stolwijk et al. reported cognitive and motor developmental delay in 23% of patients in a systematic review of 1- to 2-year outcomes using the Bayley Scales of Infant and Toddler Development (Stolwijk et al., 2016). Delays were most prominent for the children with congenital diaphragmatic hernia, generally the most unwell group of anomalies requiring non-cardiac surgery, and therefore the most vulnerable to brain injury. Similarly, the DAISy study reported reduced Bayley Scales on all domains amongst infants who had either cardiac or non-cardiac surgery in the first 90 days of life, at both 1 and 3 years of age (Walker et al., 2015).

### Risk of Brain Injury in Neonates Requiring Surgery

There are multiple potential contributors to brain injury in infants requiring neonatal surgery, beyond the direct impact of congenital anomalies, including: hypoxia and ischemia related to the underlying condition; vulnerability due to comorbid genetic anomalies; pain, sedation and analgesia; surgery and anesthesia; prolonged hospitalization; the altered family environment and impact on parental mental health, as well as parent-infant attachment (Govindaswamy et al., 2020; Diffin et al., 2016).

The literature regarding the impact of anesthesia on the neonatal brain is rapidly expanding. Whilst retrospective studies of infants have raised some concerns about anesthetic toxicity in the developing brain, prospective research has been somewhat reassuring. The GAS trial taught us that 40 min of general anesthesia for the repair of inguinal hernias in boys did not impact on measured 2- or 5-year neurodevelopmental outcomes, compared to those who received a spinal anesthetic (Davidson et al., 2016; McCann et al., 2019). A Canadian population-based study also showed no increased risk of adverse child development on school entry for children who had undergone surgery compared with sibling-matched pairs (O'Leary et al., 2019). However, that study intentionally excluded children with neurodevelopmental impairment. Of course, anesthesia and surgery occur for a few hours amongst what is often many weeks spent in the neonatal intensive care unit (NICU) for babies with congenital anomalies.

While clinical studies to date have largely focused on the impact of anesthesia on healthy infants, much pre-clinical research should lead us to interpret these findings with caution (Ben-Ari, 2002). Many anesthetic and sedative agents are GABA antagonists and have been shown to be neurotoxic in neonates (Andropoulos, 2018; Gascoigne et al., 2021). It also follows that the variability of development of GABA synapses may also further be altered by other neuronal disruptions such as prematurity. GABA is excitatory in the newborn brain, becoming inhibitory as chloride co-transporters mature, most importantly KCC2. Extensive animal research has identified the potentially toxic effects of a mismatch between GABA- and glutamate-mediated inhibition and excitation, which may occur with altered maturation of chloride co-transporters. Ben-Ari proposed that ‘the shift of GABA actions is a developmentally regulated function that signals the shift from a genetically determined programme to one that takes neuronal and environmental factors into account’.

Minimal human research exits regarding the impact of anesthesia on neonates who have already experienced some degree of brain compromise and in those requiring multiple operations over the first weeks of life. The extended peri-operative period and intensive care stay clearly exposes infants to ongoing risks beyond the surgery and anesthesia itself. For example, exposure to opiates both *in utero* and during preterm care has impacts on brain growth and later neurodevelopmental profile (Ranger et al., 2015; Zwicker et al., 2016; Steinbauer et al., 2021; Walhovd et al., 2007). Moreover, exposure to pain itself during the preterm course has an impact on thalamic development and longer-term neurodevelopment (Duerden et al., 2018; Schneider et al., 2018).

Functional deficits may also emerge through limits to prone positioning in the NICU following thoracic or abdominal surgery. The development of feeding disorders is also frequently experienced by neonates due to the balance of adverse oral experiences such as regular suctioning or severe gastro-esophageal reflux disease (Ferguson, 2017). The physical impact of impairment on normal nutritional intake for infants who are dependent on parenteral nutrition, or those with excessive caloric requirements due to underlying disease, also has the potential to impact both physical and brain development.

The risks to neonates undergoing surgery for congenital anomalies are demonstrated through imaging studies showing increased rates of brain injury and deviation from normal growth and development patterns. Moran et al. reported the post-operative MRI findings of 39 neonates with congenital diaphragmatic hernia, esophageal atresia or abdominal wall defects, who were subsequently assessed at 2 years of age (Moran et al., 2019). Compared with gender and gestational-age matched controls, infants requiring surgery had smaller brains on quantitative measures, were 10 times more likely to have white matter signal abnormalities and 6 times more likely to have delayed gyral maturation. The group who underwent surgery for congenital anomalies also had lower mean scores than controls in all three domains of the Bayley Scales at 2 years.

Stolwijk et al. performed MRI brain scans post-operatively in 101 infants, of whom 32 were born prematurely (Stolwijk et al., 2017). MRI abnormalities were found in 75% pre-term and 58% full-term infants. In the pre-term group, most abnormalities were parenchymal and included white matter lesions, punctate cerebellar lesions, thalamic infarction and periventricular hemorrhagic infarction. Prematurity and the type of congenital anomaly were most predictive of brain injury.

### Surgery and Prematurity

Congenital anomalies have long been known to be an independent risk factor for neonatal morbidity in infants born prematurely (Linhart et al., 2000). In an outcome study of 5- and 8-year-olds with abdominal wall defects, infants born before 37 weeks gestation performed worse on every test compared with term-born infants at both 5 and 8 years (Burnett et al., 2018). In infants with congenital diaphragmatic hernia, lower gestational age was an independent risk factor for impaired language and motor development at 2 years but had an ongoing impact on full-scale intelligence quotient (FSIQ) and processing speed even at 8 years (Gunn-Charlton et al., 2019). For infants born with esophageal atresia, the acute impact of comorbid prematurity is substantial, including pre-operative respiratory compromise, delayed repair and gastrostomy formation, increased risk of surgical complications, oral aversion and feeding difficulties, length of stay as well as potential comorbid lesions including cardiac anomalies (Hunt et al., 2016). In 32.2-year-olds with esophageal atresia, infants with a Bayley score more than 1 standard deviation below the test normative mean at 2 years were, on average, 3 weeks more premature, 1 kg lighter at birth and had more than twice the hospital length of stay, compared with infants with a 2-year Bayley score in the normal range (Gunn et al., 2016b). However, by school-age the impact of prematurity was overcome by other contributing illness-related and environmental factors (Burnett et al., 2021).

If surgery itself (as well as the condition leading to surgery) is a risk to term-born neonates, then the additive impact of prematurity is potentially cumulative. Hunt et al. reported the 8-year-old outcomes of three cohorts of extremely preterm infants who underwent a surgical procedure during their NICU admission (Hunt et al., 2018). They found that rates of neurosensory disability were more than 4-fold higher in children who had undergone surgery (33%) compared with those who didn’t (10%). Surgery in those infants would most likely have been for conditions such as necrotizing enterocolitis or a patent ductus arteriosus ligation rather than for management of a congenital anomaly *per se*.

Walsh et al. specifically excluded congenital anomalies from a group of preterm infants less than 30 weeks gestation included in a study comparing MRI at term-equivalent age and 2-year outcome between those who did and did not receive surgery and anesthesia (Walsh et al., 2021). A relationship was found between exposure to surgery (with a dose effect of time) and relative reductions in white matter and deep gray matter volume. At 2 years, surgical exposure was associated with lower cognitive and motor scores and there remained a dose effect of surgical time, even when controlling for the MRI findings, suggesting that more complex microstructural factors are at play beyond brain growth. Gano et al. also described adverse neurodevelopmental outcomes in preterm infants born before 33 weeks who underwent two or more surgeries during the preterm period, with 20-point lower FSIQ at school age compared with children had not undergone surgery (Gano et al., 2015).

Most major neonatal surgery is time-critical such that pre-operative MRI is very challenging to obtain. Those researchers who have managed to obtain such images are subject to extreme bias as the more complex or unwell infants are least likely to have been able to access the MRI scanner prior to surgery. Fetal imaging would be the obvious solution to exploring brain growth prior to non-cardiac surgery but has not yet been widely explored to date.

## Brain Injury in Congenital Heart Disease

Congenital heart disease (CHD) makes up the largest cohort of infants with congenital anomalies requiring neonatal surgery and has provided a helpful model to understand the impact of impaired fetal development on the growing brain. Fortunately, cardiac anomalies often have a slightly longer window of opportunity to allow pre-operative imaging than non-cardiac surgical anomalies. Furthermore, there is an extensive literature exploring fetal MRI in this population.

### Fetal and Neonatal Brain Development With CHD

Many studies using both ultrasound and MRI have now shown reductions in brain growth during the second and third trimester in fetuses with CHD, the variance from normal fetal brains of which becomes more pronounced through pregnancy (Limperopoulos et al., 2010; Ortinau et al., 2019). Early neonatal MRI confirms that brain abnormalities include delayed myelination patterns, impaired white matter microstructural development, abnormal brain metabolism, abnormal cortical folding and reductions in brain volumes (Beca et al., 2013; Ortinau et al., 2013; Ortinau et al., 2012; Miller et al., 2007; Licht et al., 2009). These findings are not specific to the cardiac diagnosis but are seen in infants with right and left heart obstruction, suggesting the impact is related more closely to chronic cerebral hypoxia rather than altered cerebral blood flow *per se*. This potential link has been confirmed by MRI studies linking cerebral oxygenation with impaired growth in fetuses with CHD (Sun et al., 2015). Gestational age and chronicity of hypoxia affect the fetal response, which most commonly includes cerebral vasodilation (Ortinau and Shimony, 2020).

It is likely that hypoxic injury in the third trimester has a similar effect on brain development to that seen in preterm infant brains. It is therefore not surprising that the pattern of injury seen in term newborns with CHD is remarkably similar that of term-corrected preterm brains. In particular, MRI studies have shown that periventricular punctuate white matter injury is very common in both populations, although the more extensive patterns of periventricular leukomalacia seen historically, and still occasionally contemporaneously, in preterm infants, are seen less commonly in CHD (Beca et al., 2013; Ortinau and Neil, 2015; Guo et al., 2019). Intriguingly, the long-term motor development trajectory varies somewhat between the two groups, with lower rates of cerebral palsy in CHD, except where later cerebrovascular accidents have occurred. However, the pattern of attentional problems into school age is very similar between the groups (Claessens et al., 2018; O'Shea et al., 2013). The differences are most likely impacted by chronicity of illness and subsequent brain impacts, especially low cardiac output syndrome and stroke.

Given the impaired fetal pattern of brain development in CHD, it follows that the brains of newborns born with CHD are more immature than their healthy counterparts (Beca et al., 2013; Licht et al., 2009). Furthermore, the brains of neonates showing evidence of immaturity relative to their gestational age on pre-operative scans, have higher rates of injury detected on post-operative MRI scans and, perhaps more importantly, are more likely to exhibit subsequent developmental delay (Beca et al., 2013; Andropoulos et al., 2014).

### Prematurity and Brain Injury in CHD

As with non-cardiac congenital anomalies, neonates with CHD are more likely than those without CHD to be born preterm and/or SGA and preterm infants are more likely to be born with CHD than those who reach full-term. Lower gestational age is a strong predictor of brain injury (encompassing a range of patterns of anomalies on neuroimaging) and neurodevelopmental impairment in CHD, even in early term-born infants. A study of 971 consecutive neonates with critical CHD was stratified by gestational age and adjusted for severity of lesion, against a reference group of neonates born at 39–40 weeks gestation. The risk of cerebral injury increased 2-fold in the 37–39 week gestation group; 8-fold in the 34–37 week gestation group and 25-fold in infants less than 34 weeks gestation at birth (Costello et al., 2010). In the *Hearts and Minds Study* of babies over 36 weeks at birth with critical CHD, on multivariable analysis of 2-year outcomes, the lowest developmental quotients on the Bayley Scales related to intrinsic patient factors including reduced gestational age at birth, single ventricle physiology with pulmonary obstruction and the need for repeat surgery (Gunn et al., 2016a). Ortinau et al. demonstrated that CHD does not increase the risk of intraventricular hemorrhage over that of gestation-associated risk (Ortinau et al., 2018), but Bell et al. showed that preterm neonates cared for in a cardiac intensive care unit are 3.2 times more likely to display acute neurological events (including cerebral hemorrhage or white matter injury) compared with older patients (Bell et al., 2019).

There has been much debate regarding optimal timing of surgery for infants born prematurely with critical CHD (Curzon et al., 2008; Hickey et al., 2012; Chu et al., 2017). Intentionally delayed surgery, beyond the best-practice timing for a given cardiac lesion, allows for organ growth and maturity, potential nutritional benefits, fall in pulmonary vascular resistance, improvement in immune competence and safe operative scheduling. Conversely, the risks of delaying surgery include the secondary effects of unrepaired CHD including hypoxemia, increased pulmonary blood flow, systemic pulmonary artery pressures, ductal steal, intensive care morbidity and the overall cost of care. Studies have shown that elective delayed surgery in preterm infants increases the risk of mortality, white matter injury on MRI and secondary morbidities such as infection, apnea, and necrotizing enterocolitis, whereas intentional delays reduce the risk of intracerebral hemorrhage, renal dysfunction and coagulopathy. These difficult decisions regarding competing clinical interests will continue to be made at an institutional level, weighing up risk and benefit for individual patients. However, it is clear than in most centers, a collaborative approach in care between cardiac intensive care, cardiology and neonatology is likely to lead to the best outcomes for preterm infants with CHD (Krishnamurthy et al., 2013).

## Conclusion

Both critical CHD and non-cardiac congenital anomalies which require neonatal surgery, increase the risk of brain injury and subsequent neurodevelopmental impairment compared with healthy infants. MRI patterns of impairment suggest different mechanisms are at play between the two surgical groups, with myelination delay more prominent in the cardiac group and gyral maturation delays more common in the non-cardiac group. However, both types of lesions place the neonate at risk of further brain injury throughout their peri-operative and intensive care course. The additive burden of prematurity is complex but combines the vulnerable states of both prematurity and relative immaturity such that an increased risk of brain injury and subsequent neurodevelopmental impairment is inevitable.

Many studies (including my own) have attempted to simplify and homogenize research findings, by excluding preterm infants from explorations of congenital anomalies on injury and outcome, when in fact this is exactly the group who needs the most attention. Rather than excluding infants with anomalies from studies of preterm neurodevelopment and preterm infants from studies of congenital anomalies, future studies should focus specifically on minimizing risk to preterm infants with comorbid congenital anomalies. This could include health-service research exploring the multidisciplinary team requirements to merge best practices across clinical craft groups with a patient-centered focus. Studies of the impact of developmental care practices in preterm infants could be extended into the surgical and cardiac environment. Further studies should provide gestation-based dosing for commonly used anesthetic and analgesic agents. Follow-up programs should incorporate later preterm infants than historically included, to allow for the potentially additive impact of surgical complexity.

A more concerted focus on these high-risk infants should obviate the increased risk of impairment through improvements in patient-centered, developmentally appropriate care, which carefully balances pain management, nutritional support and brain-protective strategies from fetal or neonatal diagnosis to childhood and beyond.
